# Lentiviral expression system for the purification of secreted proteins from human cell cultures

**DOI:** 10.1186/s12896-016-0288-3

**Published:** 2016-09-02

**Authors:** Alexander Falkenhagen, Sabah Asad, Stanley E. Read, Sadhna Joshi

**Affiliations:** 1 Department of Laboratory Medicine & Pathobiology, University of Toronto, Toronto, Canada; 2 Department of Pediatrics, The Hospital for Sick Children, Toronto, Canada; 3 Department of Molecular Genetics, University of Toronto, Toronto, Canada

**Keywords:** His-tag, Human cells, Lentiviral vector, Purification, Secreted proteins, Soluble CD4

## Abstract

**Background:**

Recombinant proteins of therapeutic use are ideally produced in human cells to ensure appropriate co- and post-translational modifications. However, purification of secreted proteins from the culture media is impeded by low expression from transfected cell lines and the presence of serum proteins. Here we describe a simple and cost-effective approach based on lentiviral vector-mediated gene delivery and expression of a secreted His-tagged protein from human embryonic kidney 293 T cells and direct affinity chromatography purification from the cell culture media.

**Results:**

Using a protein-based HIV entry inhibitor, soluble CD4 (sCD4), we demonstrated that 293 T cells transduced with a lentiviral vector mediated over 10-fold higher secretion of sCD4 in comparison to 293 T cells transfected with the corresponding plasmid. Secretion of sCD4 increased with the dose of the lentiviral vector up to a multiplicity of infection of 50. Exchanging the native signal peptide of sCD4 with the signal peptide of human alpha-1 antitrypsin increased expression by 50 %. There was no difference in expression from a monocistronic or bicistronic lentiviral vector. Reduction of the serum concentration in the culture media had no significant effect on the secretion of sCD4. Small-scale purification from 50 ml of culture media with reduced serum content yielded up to 1 mg of pure sCD4. Purified sCD4 bound to recombinant HIV envelope glycoprotein 120 (Env gp120) and inhibited HIV entry at concentrations comparable to published results.

**Conclusion:**

The procedure outlined in this study can be performed without the need for specialized reagents or equipment and could easily be adapted by any laboratory. Furthermore, the method could be used to produce sCD4 fusion proteins or other His-tagged proteins.

## Background

Expression systems based on bacteria, yeast, insect cells and plants have successfully been used to express recombinant proteins at high levels. Each system has its own advantages, but none can fully recapitulate the extensive co- and post-translational modifications found in some human proteins [1–4]. In order to ensure functionality and to be as close as possible to their native state, many human proteins are now produced in Chinese hamster ovary (CHO) cells and human embryonic kidney (HEK) cells. Both cell lines are well established and have been utilized to produce clinically relevant proteins [5]. While both CHO and HEK cells are capable of extensive glycosylations, it has recently become more and more evident that proteins produced in CHO cells have a different glycosylation pattern than the same proteins produced in HEK cells, which may affect their function [6–10]. For example, a fusion protein containing the extracellular domain of the human interleukin-23 receptor and a crystalizing fragment (Fc) region produced in HEK cells was less stable in mice than the same protein produced in CHO cells [6]. In another study, an Fc receptor (CD16) produced in HEK cells exhibited slower antibody binding and dissociation kinetics in comparison to CD16 from CHO cells [7]. While the exact implications of differences in glycosylation are not yet fully understood, a strong argument for the production of therapeutic proteins in HEK cells is made by the finding that CHO cells can add terminal galactose-α-1,3-galactose (α-Gal) to proteins [11, 12]. α-Gal is a non-human antigenic glycan and its reaction with circulating antibodies present in most individuals can lead to anaphylaxis [11, 13]. This glycan is absent in proteins produced in HEK cells [14].

Production of secreted proteins in human cells is usually achieved by large-scale transient transfections to compensate for low expression levels. However, repeated large-scale transfections require high quantities of a transfection reagents and plasmid DNAs. Highly efficient transfection systems with specialized reagents and cell lines are available, but are commonly substituted with polyethylenimine (PEI) transfection of HEK cells to reduce the cost [15–17]. An alternative to transient transfection is the generation of stable clonal cell lines [18]. Stable chromosomal integration and expression from transfected DNA relies on coupling protein expression to a selectable marker. Since transgene expression varies depending on the integration site, different clones are isolated by limiting dilution and tested for transgene expression, which is tedious. Transduction with lentiviral vectors is another method of generating stable cell lines. Cells transduced with lentiviral vectors have been used to express secreted proteins [19, 20]. However, the lentiviral vector backbones used in these studies were custom made and are not readily available. Furthermore, production and titration of these vectors required specific cell lines, media, and lengthy concentration procedures [19, 20]. Regardless of the expression system, the recombinant proteins are usually purified from ≥1 l cell culture supernatants. Since standard culture media contain high amounts of serum proteins that can interfere with the purification process or contaminate the final protein preparations [21], serum proteins are removed prior to protein purification or specialized serum-free media is used.

In order to facilitate the purification process, the protein of interest is usually fused to a protein tag (e.g. human influenza hemagglutinin). Immobilized antibodies directed against the protein tag are commonly used to isolate the protein of interest from the culture media [15].

Here we describe a simple and effective method for the purification of a His-tagged human protein from the culture media of lentiviral vector-transduced HEK 293 T cells. All reagents are readily available at a relatively low cost and no specialized equipment is needed to complete the procedure. Using soluble CD4 (sCD4), a truncated version of the CD4 receptor that is secreted, we were able to purify up to 1 mg of the protein from small-scale cell cultures. The expression system and the purification method used here can easily be adapted for purification of other proteins by most laboratories.

## Methods

### Cells, plasmids and proteins

HEK 293 T cells were obtained from Dr. Jason Moffat (University of Toronto, Toronto, Canada). 293 T and TZM-bl cells were cultured in Dulbecco’s modified Eagle’s medium (DMEM; Thermo Fisher Scientific, Waltham, United States) supplemented with 10 % fetal bovine serum (FBS, Thermo Fisher Scientific) and 1 % Antibiotic-Antimycotic (Thermo Fisher Scientific). The self-inactivating bicistronic lentiviral vector pLV-CMV was constructed by replacing the *Pst*I-to-*Nhe*I fragment of pLVX-IRES-ZsGreen1 (Clontech, Mountain View California, United States) with the compatible *Nsi*I-to-*Avr*II fragment from pLJM1-EGFP [22]. The monocistronic vector pLV-CMV-M was generated by deleting the *Sac*II fragment of pLV-CMV. The gene encoding sCD4 (amino acids 1-209 of CD4, UniProtKB P01730, followed by GGGSGAGCCPGCC HHHHHH) and the different signal peptides were synthesized and sequenced by Genscript (Piscataway, United States). The gene encoding sCD4 was preceded by an *Eco*RI site and followed by a *Not*I site and cloned as an *Eco*RI-to-*Not*I fragment into the multiple cloning site of the bicistronic lentiviral vector pLV-CMV to generate pLV-CMV-sCD4. The nucleotide sequence of the signal peptides was flanked by an *Eco*RI site at the 5’ end and nucleotides 76-218 of the open reading frame of sCD4 containing an *Apa*I site at the 3’ end. The *Eco*RI-to-*Apa*I fragments containing the different signal peptide sequences were each used in tripartite ligation reactions with the *Apa*I-to-*Bam*HI and *Bam*HI-to-*Eco*RI fragments of pLV-CMV-sCD4 to generate pLV-CMV-AAT-sCD4, pLV-CMV-TPA-sCD4 and pLV-CMV-IgG-sCD4. The *AAT*-*sCD4* gene was transferred from pLV-CMV-AAT-sCD4 as an *Nde*I-to-*Not*I fragment to the corresponding sites of the monocistronic vector pLV-CMV-M to generate pLV-CMV-M-AAT-sCD4. The pJRFL-env and pHXB2-env plasmids expressing HIV Env gp160 from HIV_JRFL_ and HIV_HXB2_, respectively, were a kind gift from Dr. Donald Branch (University of Toronto). The following reagents were obtained through the NIH AIDS Reagent Program, Division of AIDS, NIAID, NIH: psPAX2 and pMD2.G from Dr. Didier Trono; TZM-bl and pSG3^Δenv^ from Drs. John C. Kappes and Xiaoyun Wu [23–28]; sCD4-183 from Pharmacia Inc. [29]; and HIV_BaL_ gp120.

### Production and titration of lentiviral vector particles

On day one, 7 × 10^6^ 293 T cells were seeded into a 10 cm cell culture dish in 9 ml culture media. 24 h later, the media were replaced with 9 ml fresh media. 1.5 h later, chloroquine (Bioshop, Burlington, Canada) was added at a final concentration of 25 μM. 10.5 μg of the lentiviral vector construct, 7 μg of psPAX2 and 3.5 μg of pMD2.G were added to a 1.5 ml tube in a final volume of 450 μl. 50 μl of 2.5 M calcium chloride were added dropwise and the tube was vortexed for a short time. After 5 min, 500 μl of 2xHBS (50 mM HEPES, 1.5 mM disodium hydrogen phosphate, 280 mM sodium chloride, 10 mM potassium chloride, and 12 mM sucrose) were added dropwise to form calcium phosphate-DNA precipitate. After vortexing the tube for a short period, the mixture was incubated for 1 min and added dropwise to the cells. The next day, media were exchanged with 10 ml fresh media and the cells were incubated for 48 h. Culture supernatants were harvested, filtered (0.45 μm pore size), aliquotted and stored at -80 °C. To determine the titer, 2 × 10^5^ 293 T cells were mixed with serial dilutions of the vector particles in the presence of polybrene (final concentration 8 μg/ml; Sigma Aldrich, Missouri, Unites States) in a 12-well plate in a final volume of 1 ml. The percentage of cells that emitted green fluorescence was determined by flow cytometry or fluorescence microscopy after 3 days.

### Transfection and transduction of 293 T cells

For transfection, 7×10^5^ 293 T cells per well were cultured in 1 ml culture media in a 12-well plate for 1 day. Media were exchanged and 1 h later each well was transfected with 0.75 μg of pLV-CMV-AAT-sCD4 using PolyJet transfection reagent (FroggaBio, North York, Canada) according to the manufacturer’s instructions. For transduction, 1×10^5^ 293 T cells were mixed with the lentiviral vector at the indicated multiplicity of infection (MOI) and polybrene (final concentration 8 μg/ml) in a 12-well plate in a 1 ml volume. The cells were incubated for 3–4 days. The cells were sub-cultured for 1–2 weeks and then used in subsequent experiments.

### PCR

Genomic DNA was extracted using the PureLink gDNA mini kit (Thermo Fisher Scientific) according to the manufacturer’s instructions. The primer sequences for the detection of integrated vector were A005-F 5’-TCTGTAGCGACCCTTTGCAG-3’ and A005-R 5’-CACGCCGTAGAACTTGGACT-3’. The primer sequences for the detection of genomic ß-actin were A006-F 5’-CAGCAAGCAGGAGTATGACGA-3’ and A006-R 5’- CACTCTGGGTAAG GACAAGTTGG-3’. The primers were synthesized by ACGT DNA Technologies Corporation (Toronto, Canada). PCRs were set up using 2X Taq FroggaMix (Froggabio), 0.5 μg genomic DNA, and 1.5 μM forward and reverse primers. The cycles were: 1) 94 °C for 10 min; 2) 94 °C for 0.5 min; 3) 60 °C for 0.5 min; 4) 72 °C for 0.5 min; and 5) 72 °C for 10 min. Cycles 2–4 were repeated 24 times.

### Detection and purification of His-tagged proteins

Culture media were clarified by short centrifugation at low speed. His-tagged proteins in the culture media were detected using a competitive Enzyme-linked immunosorbent assay (ELISA; Genscript) according to the manufacturer’s instructions. Briefly, culture media and a mouse anti-His-tag mAb were added to wells coated with a His-tagged protein. The wells were washed and bound mAb was detected with an horseradish peroxidase (HRP)-conjugated goat anti-mouse IgG. Following additional washes, HRP activity was visualized using 3,3′,5,5′-tetramethylbenzidine substrate and the reaction was stopped. SDS-PAGE, Coomassie blue staining, and Western blot analyses were performed as described previously [30, 31]. Some gels were stained using One-Step Blue Protein Gel Stain (Biotium, Hayward, United States). For purification, a HisTalon cobalt gravity column (Clontech) was equilibrated with 10 ml equilibration buffer (50 mM sodium phosphate, 300 mM sodium chloride; pH 7.4). 50 ml of the clarified culture media were directly applied to the column at room temperature. The column was washed with 8 ml equilibration buffer and 7 ml wash buffer (50 mM sodium phosphate, 300 mM sodium chloride, 10 mM imidazole; pH 7.4). The proteins were eluted with 4 ml elution buffer (50 mM sodium phosphate, 300 mM sodium chloride, and 150 mM imidazole; pH 7.4) in 3 fractions (first fraction, 1 ml; second fraction 1.5 ml; and third fraction 1.5 ml). The second fraction contained the majority of the protein and was applied to a 5 ml Zeba spin column with 7 K MWCO (Thermo Fisher Scientific) to exchange the elution buffer with PBS.

### HIV gp120 binding assay

Purified HIV_BaL_ gp120 (2 μg/ml; 100 μl per well) was diluted in 0.05 M sodium carbonate buffer, pH 9.6 and coated onto untreated 96-wells overnight at 4 °C. Uncoated wells served as a control. Wells were washed 4 times with PBS containing 0.05 % Tween 20 (Sigma), blocked with PBS containing 5 % bovine serum albumin fraction V (BSA, Bioshop) for 2 h at room temperature, followed by 4 washes. 50 μl of sCD4 diluted in PBS containing 0.1 % BSA and 0.05 % Tween 20 were added to all wells at the indicated concentrations and incubated overnight at 4 °C. Wells were washed 4 times and incubated with 50 μl of a ready-to-use mouse anti-His-tag monoclonal antibody solution (Genscript) for 1 h at room temperature. After 4 additional washes, 100 μl of a ready-to-use HRP-conjugated goat anti-mouse IgG solution (Genscript) were added to each well and the plates were incubated for 30 min. After 4 washes, 100 μl of 3,3',5,5'-Tetramethylbenzidine substrate (Genscript) were added for 5 min and the reaction was stopped by adding Stop Solution (Genscript). The OD at 450 nm was determined using a standard plate reader and background values obtained for control wells were subtracted.

### Antiviral assay

Replication-incompetent virus was generated by transfecting 293 T cells with 10.5 μg of pSG3^Δenv^ encoding the HIV genome with a deletion in the HIV Env gp160 open reading frame and 10.5 μg of an HIV Env gp160-encoding plasmid (pHXB2-env, pJRFL-env or pMD2.G) using the calcium phosphate transfection method described above for lentiviral vector particle production. The 50 % tissue culture infectious dose (TCID50) and inhibition of HIV entry were determined in TZM-bl cells, which express the *lacZ* gene under the control of the HIV long terminal repeat promoter. Virus (5×10^3^ TCID50) was mixed with sCD4 at the indicated concentration and added to 5 × 10^4^ TZM-bl cells together with polybrene (8 μg/ml final concentration) in 24-well plates. The cells were incubated for 2 days, fixed with 1 % formaldehyde for 5 min, and stained with X-Gal (0.4 mM potassium ferricyanide, 0.4 mM potassium ferrocyanide, 20 mM magnesium chloride, 0.4 μg/ml X-Gal) overnight at room temperature. Three random microscope images were taken for each well and infected (blue) cells were counted.

### Statistical analysis

Data are presented as means +/− standard error of the mean. Where appropriate, unpaired *t* test was used to test for statistical difference between two groups.

## Results and discussion

### Lentiviral vector expressing sCD4 and transduction of HEK 293 T cells

**Fig. 1:**
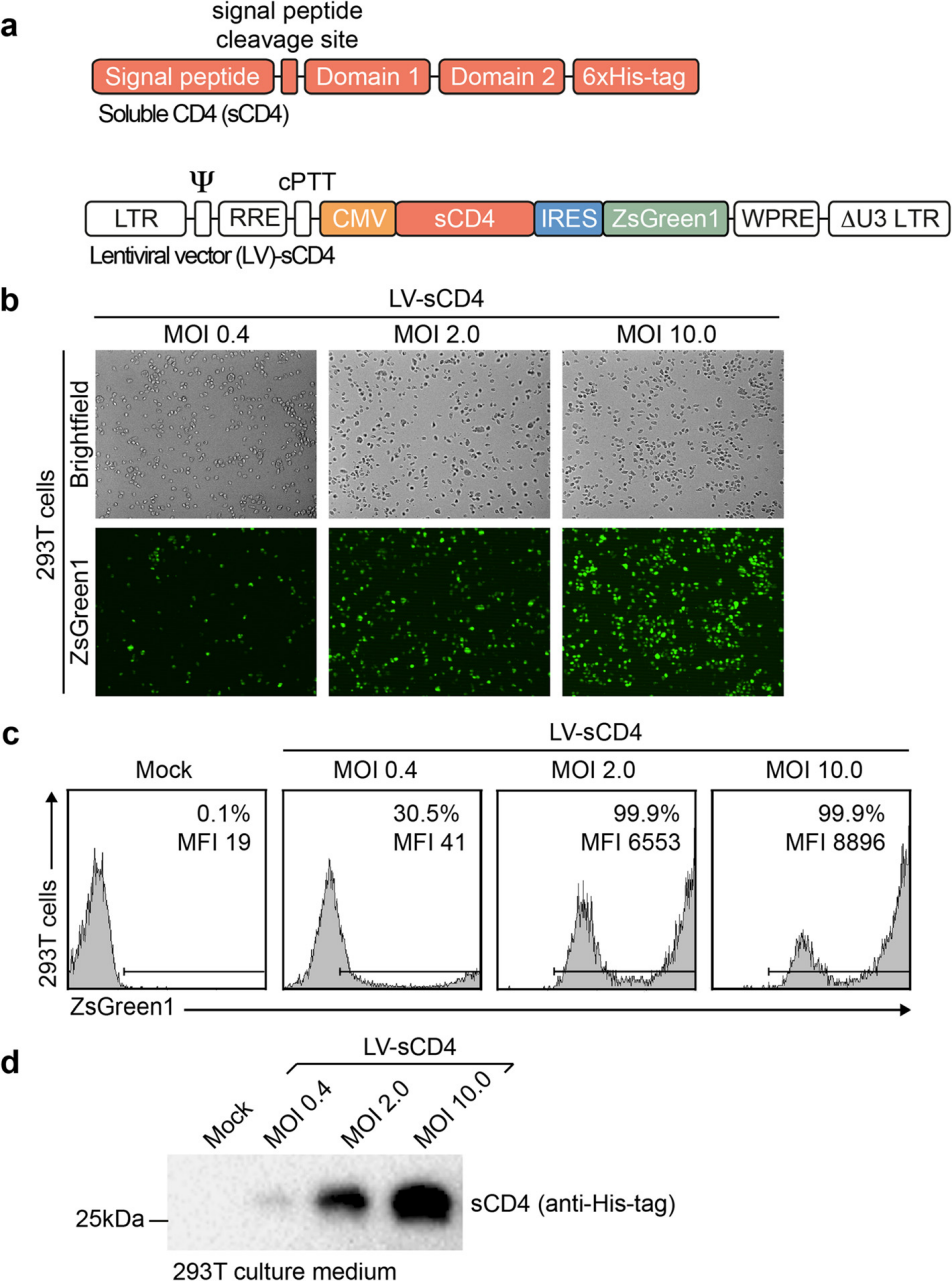
Lentiviral transduction of HEK 293 T cells. **a** Schematic diagram of the sCD4 open reading frame and the bicistronic lentiviral vector used in this study. LTR = long terminal repeat; Ψ = packaging signal; RRE = rev response element; cPPT = central polypurin tract; IRES = internal ribosome entry site; ΔU3 LTR = LTR with a deletion in the U3 promoter/enhancer region. **b**-**d** 293 T cells were transduced with LV-CMV-sCD4 at the indicated MOIs. 2 × 10^5^ gene-modified 293 T cells were seeded in a 12-well plate in 1 ml media and grown for 4 days. The cells were trypsinized and analyzed by (**b**) fluorescence microscopy and (**c**) flow cytometry. **d** Supernatants were collected, centrifuged, and analyzed by Western blot using an anti-His-tag antibody. MFI = median fluorescence intensity. The results are representative of at least 2 independent experiments

sCD4 mimics the natural CD4 receptor that is necessary for HIV entry into target cells. The secreted protein is the first HIV entry inhibitor described in the literature [32]. Administration of high doses of sCD4 produced in CHO cells has been shown to reduce viral load in two small clinical trials [33, 34]. However, frequent injections of highly purified proteins were not feasible for the long-term treatment of HIV-infected individuals. Currently, sCD4 is used in countless studies examining the mechanism and inhibition of HIV entry. The open reading frame of the *sCD4* gene used in this study is comprised of the native signal peptide and the first two domains of CD4, which allow binding to the HIV Env gp120. The second domain is followed by a 6xHis-tag. A schematic of sCD4 is shown in Fig. 1a. While the His-tag may be less effective than newer tags, it is one of the most widely used tags and antibodies as well as resins for affinity chromatography are available at a relatively low cost.

We chose to express sCD4 in HEK 293 T cells because of their human origin and availability in many laboratories. We used a self-inactivating lentiviral vector, LV-CMV-sCD4, to minimize activation of neighboring genes [35]. The vector contained the human cytomegalovirus immediate early (CMV) promoter and is highly active in 293 T cells [36]. The bicistronic mRNA transcribed from this vector allows simultaneous expression of sCD4 and the enhanced fluorescent reporter protein ZsGreen1 [37]. Additionally, the vector contained the woodchuck hepatitis post-transcriptional regulatory element (WPRE), which has been shown to increase both the titer and transgene expression in conjunction with the CMV promoter [38, 39]. Similar vectors are readily available at any plasmid repository. A schematic of the vector is shown in Fig. 1a.

Calcium phosphate transfection of 293 T cells resulted in sufficiently high vector titers without the need for further concentration of vector particles. 293 T cells showed a vector dose-dependent increase in the expression of ZsGreen1. Both the percentage of transduced cells and fluorescence intensity increased with higher doses as shown by fluorescence microscopy and flow cytometry (Fig. 1b and c, respectively). Simultaneously, the concentration of sCD4 in the culture supernatant of transduced 293 T cells increased with the vector dose (Fig. 1d).

### Utilizing heterologous signal peptides to increase secretion

**Fig. 2:**
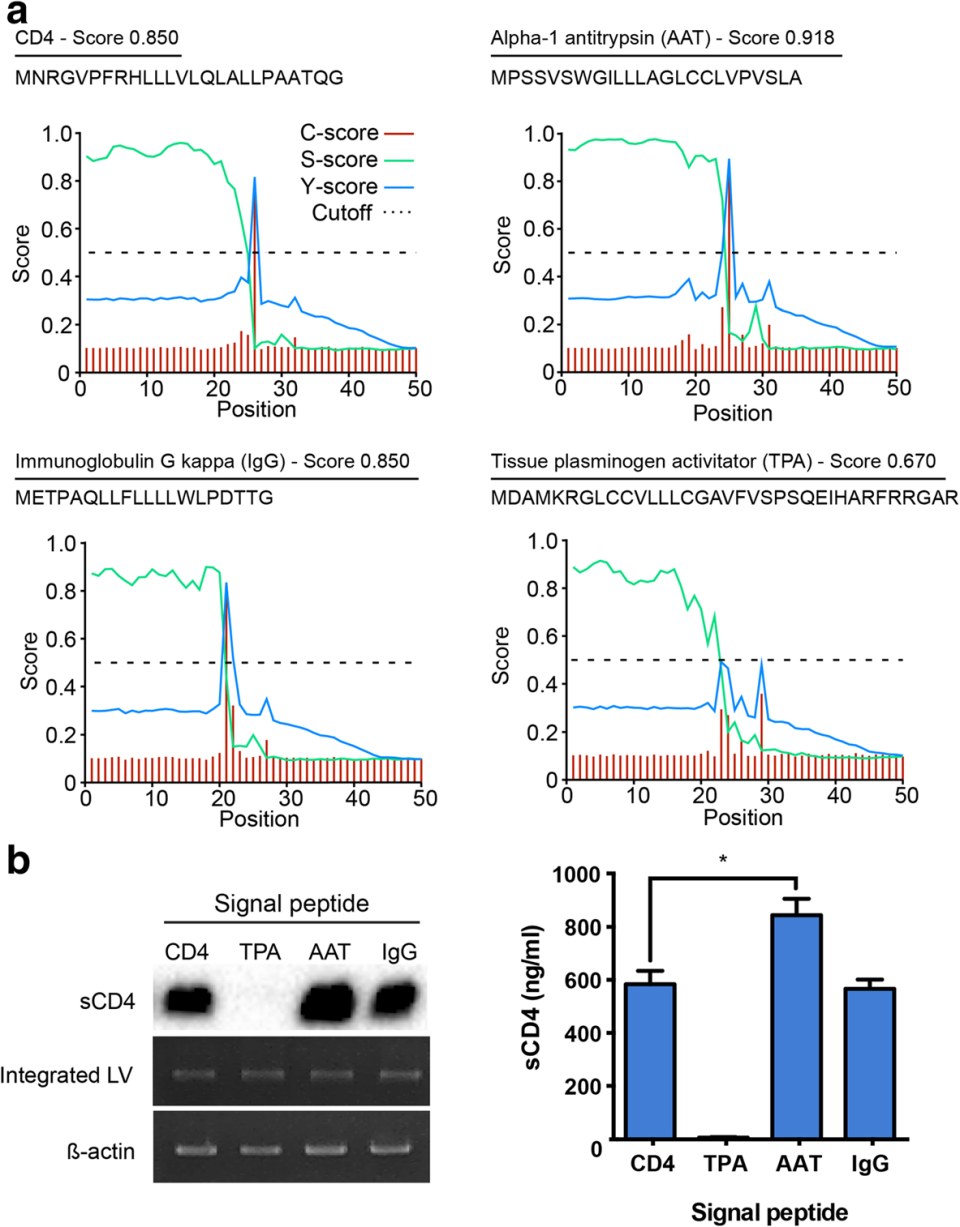
Utilizing alternative signal peptides to optimize protein secretion. **a** The signal peptide prediction software SignalP was used to analyze sCD4 with different signal peptide sequences. The C-score identifies the signal peptide cleavage site. The S-score distinguishes signal peptides from the mature protein. The Y-score is a combination of the C- and S-scores. The signal peptides, their sequence, and their final score are indicated. **b** 293 T cells were transduced with LV-CMV-sCD4 (CD4), LV-CMV-TPA-sCD4 (TPA), LV-CMV-AAT-sCD4 or LV-CMV-IgG-sCD4 (IgG) encoding sCD4 with the indicated signal peptide at a MOI of 2. 2 × 10^5^ gene-modified 293 T cells were seeded in a 12-well plate in 1 ml media and cultured for 4 days. Genomic DNA was extracted from the cells and examined for the presence of integrated vector by PCR with vector-specific primers. PCR with β-actin primers was performed as a control for equal input DNA (*left panel*). Supernatants were collected, centrifuged, and analyzed by Western blot using an anti-His-tag antibody (*left panel*) and His-tag ELISA (*right panel*). The results are representative of 2 independent experiments performed in duplicates; *p < 0.05

The signal peptide is responsible for directing proteins to the secretory pathway. Altering the signal peptide sequence has previously been shown to increase secretion for some proteins [40, 41]. We exchanged the natural signal peptide of sCD4 with three commonly used signal peptides and analyzed the resulting amino acid sequences with the bioinformatics program SignalP, which discriminates secreted from non-secreted proteins [42]. The signal peptides and their SignalP scores are depicted in Fig. 2a. The highest score was obtained for sCD4 with the alpha-1 antitrypsin (AAT) signal peptide. The score for sCD4 with the immunoglobulin G kappa chain signal peptide (IgG) was same as the score for sCD4 with the native signal peptide. sCD4 with the signal peptide of the tissue plasminogen activator (TPA) yielded the lowest score. The *sCD4* genes with the different signal peptides were cloned into the lentiviral vector and the resulting vectors were used to transduce 293 T cells. The percentage of gene-modified cells was >90 % for all vectors at a MOI of 2 (data not shown). The AAT signal peptide mediated a 50 % increase in protein concentration, while the TPA signal peptide caused a decrease, and the IgG signal peptide no significant change (Fig. 2b). Semi-quantitative PCR analysis of genomic DNA from these cells indicated that the observed differences in sCD4 secretion levels were not due to varying copy numbers of the integrated proviral vector DNA (Fig. 2b). The protein concentration in the culture media seemed to correlate with the predicted Signal P scores, e.g. the sCD4 with the AAT signal peptides had the highest score and was present at the highest concentration. Since the AAT signal peptide mediated a significant increase over the native CD4 signal peptide, all subsequent experiments were performed with sCD4 containing the AAT signal peptide.

### HEK 293 T cells can be transduced at high MOIs

**Fig. 3:**
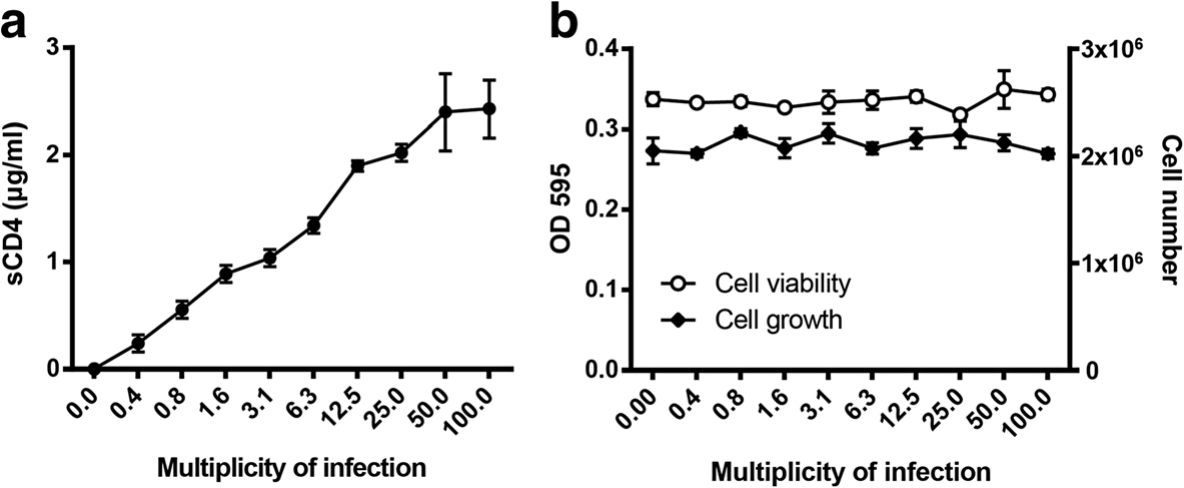
Human 293 T cells can be transduced at high MOIs. 293 T cells were transduced with LV-CMV-AAT-sCD4 at the indicated MOIs. 2 × 10^5^ gene-modified 293 T cells were seeded in a 12-well plate in 1 ml media and cultured for 4 days. **a** Concentration of sCD4 in the culture supernatants determined by His-tag ELISA. **b** Cell growth analyzed by microscopy and cell viability determined by MTT-assay. The results are representative of at least 2 independent experiments

293 T cells are an easy to transduce cell line and close to 100 % transduction efficiency can be achieved at relatively low MOIs. In order to determine the maximal vector dose that can be used, 293 T cells were transduced with LV-CMV-AAT-sCD4 at MOIs ranging from 0.4 to 100. Although reduced cell viability was evident immediately after transduction at a MOI of 100, all cells recovered after a week (data not shown). The sCD4 concentration in the media increased up to a MOI of 50 (Fig. 3a). No differences in cell growth or viability were evident in comparison to untransduced control cells (Fig. 3b). Therefore, cells transduced at a MOI of 50 were used in the following experiments.

### Optimizing conditions for the purification of sCD4

**Fig. 4:**
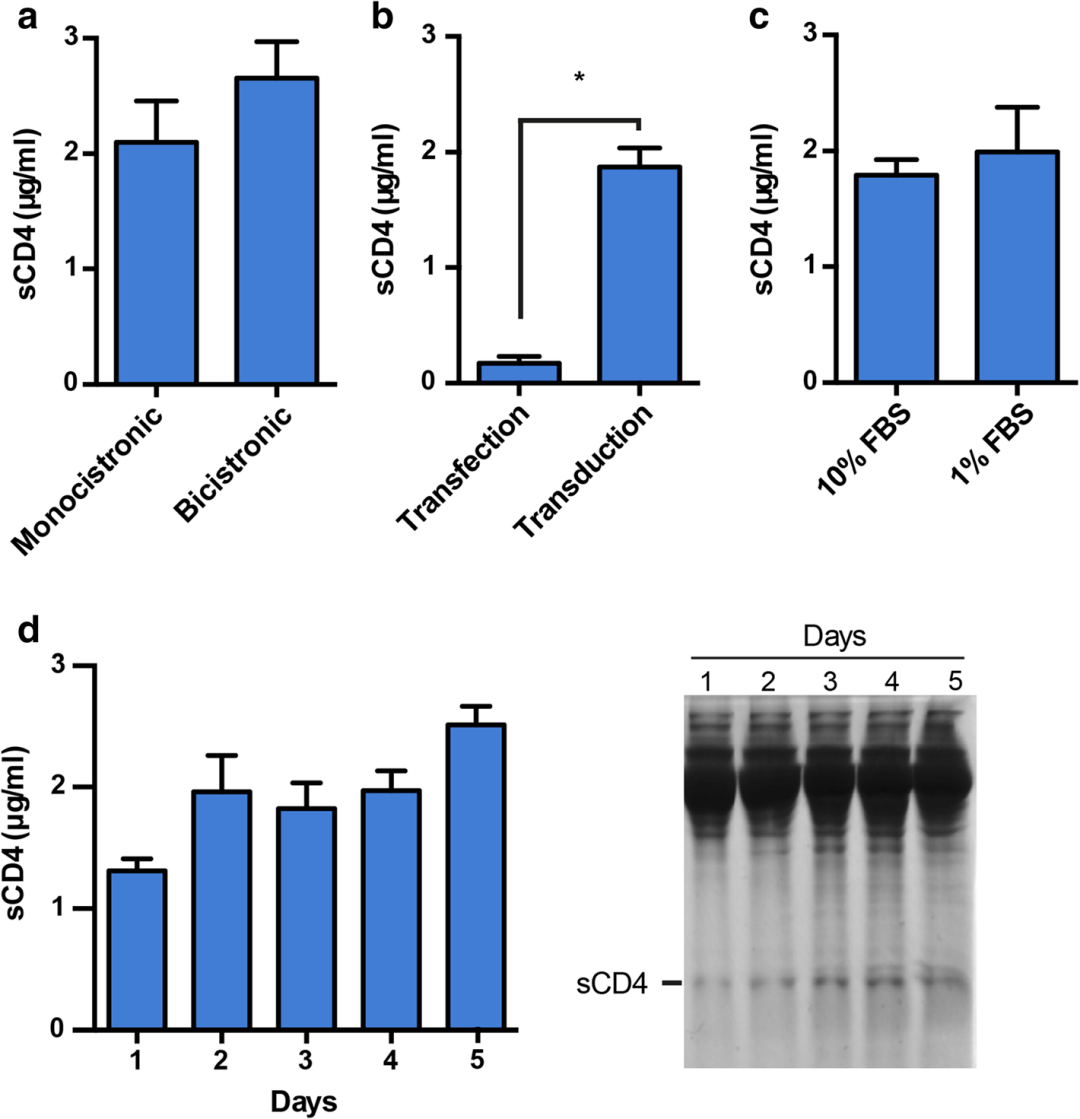
Optimizing conditions for the purification of sCD4. **a** Monocistronic versus bicistronic vector design. Cells were transduced with the bicistronic vector LV-CMV-AAT-sCD4 or the monocistronic vector LV-CMV-M-AAT-sCD4 at a MOI of 50. 2 × 10^5^ gene-modified 293 T cells in 1 ml media were plated in a 12-well plate and cultured for 4 days before culture supernatants were analyzed by ELISA. **b** Effect of stable versus transient expression on sCD4 secretion levels. 7 × 10^5^ untransduced 293 T cells or 293 T transduced with LV-CMV-AAT-sCD4 were plated in a 12-well plate in 1 ml media and cultured for 1 day. Media were replaced with fresh media and unmodified 293 T cells were transfected with pLV-CMV-AAT-sCD4. On day 2 after plating, media were replaced with fresh media and the cells were cultured for 2 additional days before ELISA was performed on culture supernatants; *p < 0.05. **c** Effect of reducing serum concentration. Gene-modified 293 T cells were cultured and analyzed as in (**b**), but on day 2 after plating culture media were replaced with media with the indicated FBS concentration. **d** Effect of incubation time. Cells were cultured as in (**c**). Media were replaced on day 2 after plating with media containing 1 % FBS and supernatants were analyzed at the indicated time points by ELISA (*left panel*) or SDS-PAGE followed by Coomassie blue staining (*right panel*). All results are representative of 2 independent experiments performed in duplicates

Lentiviral vector design can significantly affect transgene expression. For example, clear differences between monocistronic and bicistronic lentiviral vectors have been shown to exist between different cell types [43]. We generated a monocistronic vector, LV-CMV-M, by removing the ZsGreen1 open reading frame from pLV-CMV. The titer of the monocistronic vector encoding sCD4 with the AAT signal peptide, LV-CMV-M-AAT-sCD4, was determined using HIV p24 antigen ELISA because it did not encode ZsGreen1. 293 T cells were transduced at a p24 concentration equaling a MOI of 50 for the bicistronic vector encoding sCD4 with the AAT signal peptide, LV-CMV-AAT-sCD4. When sCD4 expression from cells transduced with the mono- or bicistronic vector was compared in parallel, no significant difference in protein concentration in the culture media was detected (Fig. 4a). This result showed that the bicistronic vector design did not have a negative effect on the expression of sCD4 under the tested conditions. Consequently, a bicistronic vector was used in the following experiments. However, if larger transgenes are used, a monocistronic vector may be used because the titer of lentiviral vectors can drop with the increased vector size.

Transient expression is commonly used to produce secreted proteins in mammalian cells. Using a commercially available transfection reagent, we compared sCD4 expression from 293 T cells transfected with the plasmid pLV-CMV-AAT-sCD4 to sCD4 expression from 293 T cells transduced with the lentiviral vector LV-CMV-AAT-sCD4. Transfected and transduced 293 T cells were cultured under the same conditions. Importantly, the sCD4 concentration in the culture supernatants from transduced 293 T cells was over 10-fold higher than in the supernatants from transfected 293 T cells (Fig. 4b). This result indicated that expression of secreted proteins from transduced cells was advantageous not only in terms of convenience, but also protein yield.

Standard culture media contain high quantities of serum proteins, which are undesirable for the purification of recombinant proteins. In our experience, 293 T cells can tolerate low levels of serum once they reach a confluent monolayer. To examine if reducing the serum concentration in standard media affects the concentration of secreted protein, transduced cells were grown to confluence and fresh media with a 10-fold reduced serum concentration were added. Figure 4c shows that reducing the serum concentration did not negatively affect sCD4 secretion. Accordingly, simply using standard culture media with reduced serum presents a cost-effective approach to reduce potential contaminants.

In an experiment to evaluate the effect of incubation time, cells were grown to confluence and reduced serum media were added. Culture supernatants were collected after 1-5 days of culture and then analyzed for the sCD4 concentration by ELISA and SDS-PAGE. ELISA results showed that the sCD4 concentration was the highest after 5 days of incubation (Fig. 4, *left panel*). However, Coomassie blue staining of an SDS-PAG showed the appearance of additional bands after 3 days, including a band corresponding to a slightly higher molecular weight protein than sCD4 (Fig. 4, *right panel*), which could represent sCD4 with an uncleaved signal peptide and indicate cell leakage of cellular proteins into the culture media. Therefore, 2 days of incubation after the media change were considered optimal for sCD4 production. Nevertheless, the incubation time could be increased, if the secreted protein is stable and a higher yield is desired, albeit potentially at the cost of purity.

### Purification of sCD4

**Fig. 5:**
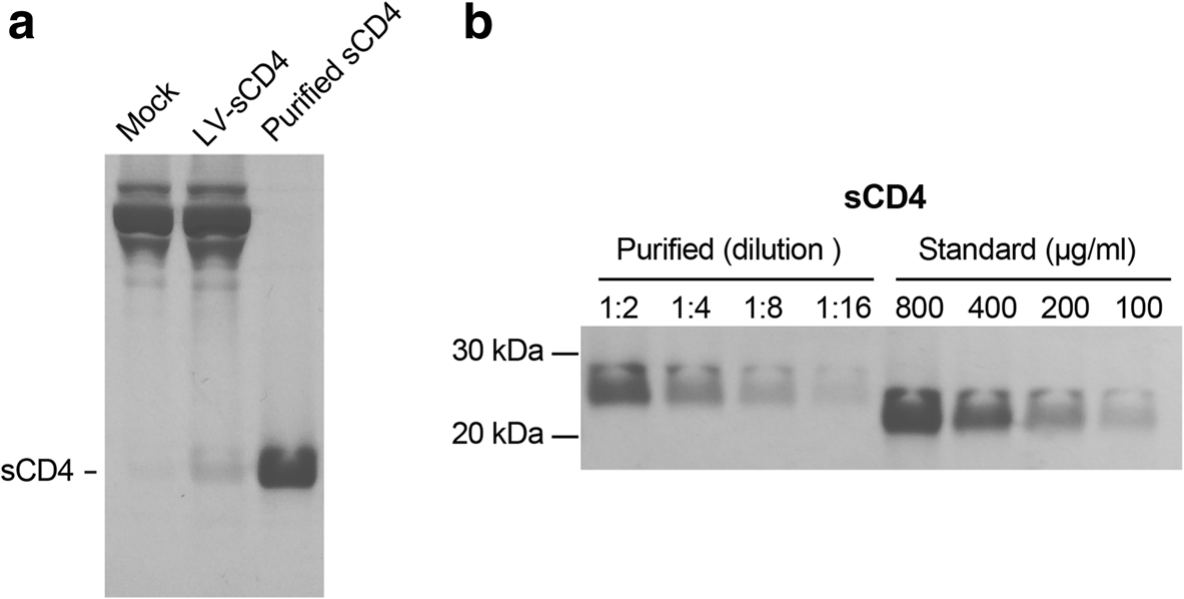
Affinity purification of sCD4. **a** SDS-PAGE analysis of purified sCD4. 7 × 10^6^ gene-modified 293 T cells were seeded into five 10 cm dishes. Media were exchanged to fresh media 1 day after plating. On the second day, media were replaced with 10 ml fresh media containing 1 % FBS and the cells were incubated for 2 additional days before sCD4 was purified from the supernatants. Samples from unmodified cells (mock), gene-modified cells (LV-sCD4) and purified sCD4 were analyzed by SDS-PAGE followed by Coomassie blue staining. **b** Comparison of purified sCD4 to an sCD4 standard from the National Institutes of Health. Equal volumes of the standard and purified sCD4 were analyzed by SDS-PAGE followed by Coomassie blue staining. Results are representative of 3 independent experiments

For affinity chromatography purification of the protein, cells were grown in five 10 cm cell culture dishes. Culture supernatants (50 ml) from transduced cells grown in reduced serum media were clarified by centrifugation and directly applied to a cobalt column. His-tagged protein was eluted and desalted. The purified protein was analyzed by SDS-PAGE, followed by Coomassie blue staining. The final product contained sCD4 devoid of any major contaminating bands (Fig. 5a). The concentration of purified sCD4 was 48 μg/ml, as determined by ELISA. However, in preliminary HIV entry inhibition assays, the antiviral activity of purified sCD4 was over 10-fold higher than expected based on previously published results (data not shown). Consequently, we compared our purified sCD4 to an sCD4 protein standard with a known concentration. SDS-PAGE analysis showed that the actual concentration was closer to 800 μg/ml, about 16.5-fold higher than the ELISA result suggested (Fig. 5b). Therefore, care should be taken if ELISA is used to determine the protein concentration.

### Purified sCD4 specifically inhibits HIV entry

**Fig. 6:**
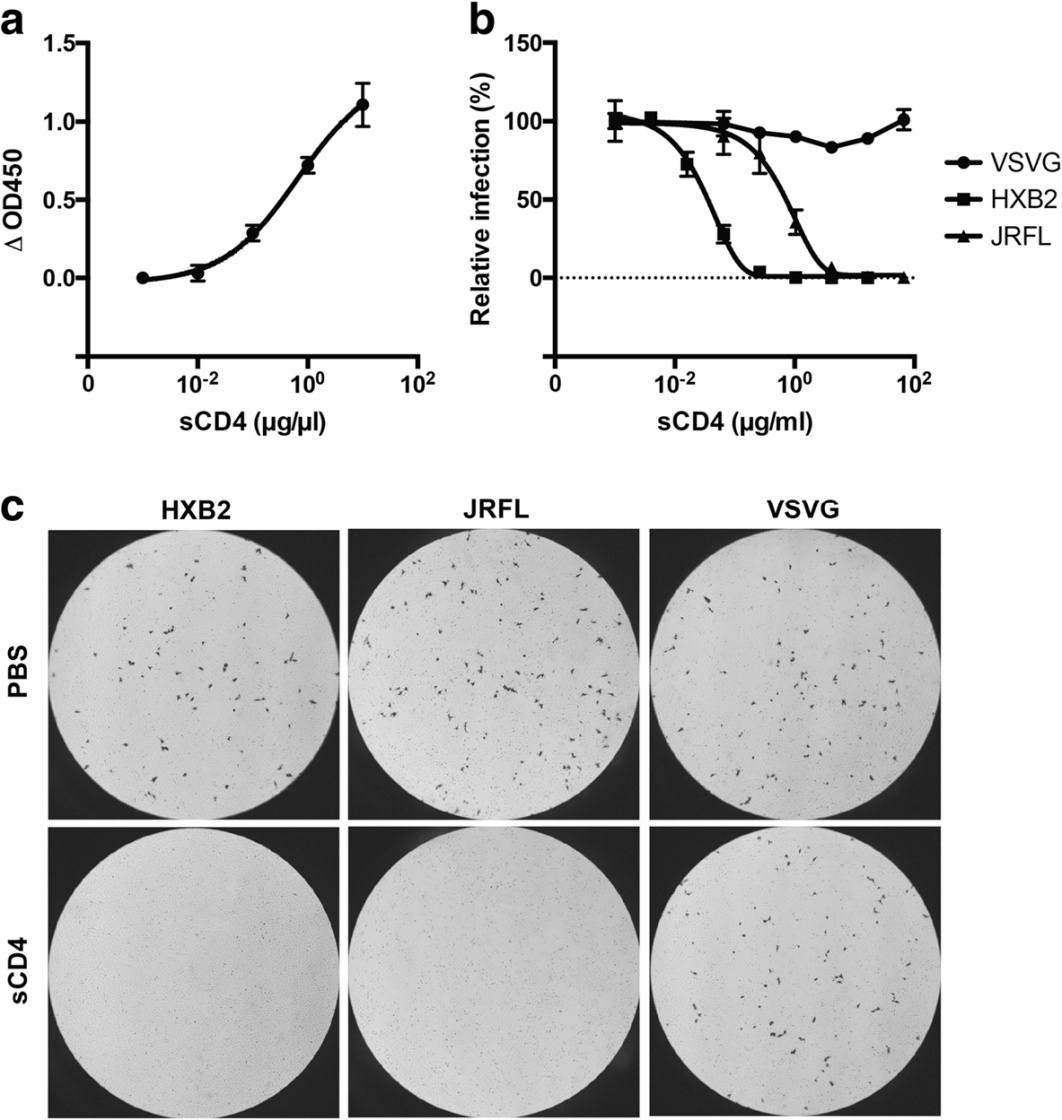
Antiviral effect of purified sCD4. **a** Binding of sCD4 to gp120. **b** Inhibition of HIV entry. 5 × 10^4^ TZM-bl cells were infected with replication-incompetent virus pseudotyped with the indicated HIV Env (TCID50 of 5 × 10^3^) in the presence of the indicated concentration of sCD4. On day 2 post-infection, β-galactosidase activity was determined by incubation with X-gal. For each sample, the number of infected cells was determined by counting the blue cells from 3 random microscopy images. **c** Representative microscopy images of cells infected in the presence (16 μg/ml) or absence of sCD4. The results are representative of 2 independent experiments

The functionality of sCD4 was assessed in an HIV Env gp120-binding assay and by inhibition of HIV entry (Fig. 6). sCD4 bound to immobilized gp120 in a dose-dependent manner (Fig. 6a). Entry of HIV strains was examined in a single-round infection assay. Replication-incompetent HIV particles were pseudotyped with the HIV_HXB2_ or HIV_JRFL_ Env gps or the unrelated VSV-G. Both HIV strains need CD4 as the primary receptor, but HIV_HXB2_ utilizes CXCR4 as a co-receptor and HIV_JRFL_ uses CCR5 as a co-receptor. In contrast, VSV-G requires the low-density lipoprotein receptor for entry. The pseudoviruses were mixed with sCD4 and added to the CD4^+^CXCR4^+^CCR5^+^ TZM-bl cells, which express the *lacZ* gene under control of the HIV Tat protein. On day 2 post-infection, cells were stained with a ß-galactosidase substrate (X-Gal) to visualize infected cells. Infection by both of the HIV strains was inhibited in a dose-dependent manner, while VSV-G entry remained unaffected (Fig. 5b). Consistent with the literature, HIV_HXB2_ was more sensitive to inhibition by sCD4 than HIV_JRFL_ (Fig. 6b and Table 1). Figure 6c shows representative microscopy images of infected wells. The half maximal inhibitory concentrations (IC50s) obtained in our experiments were comparable to previously published results (Table 1), confirming that concentrations obtained by ELISA underestimated the actual protein concentration.

**Table 1:** Protein concentration and IC50s

Method	Protein concentration (μg/ml)				IC50 (μg/ml)		CATNAP IC50 (μg/ml)	
	Purification 1	Purification 2	Purification 3	Mean ± SEM	HXB2	JRFL	HXB2	JRFL
ELISA	48	82	69	66 ± 10	0.002	0.042	0.023	1.520
Adjusted	800	1353	1133	1095 ± 160	0.032	0.684		

The sCD4 concentrations of purified preparations as determined by ELISA and the adjusted concentrations in relation to an sCD4 standard are shown. The corresponding IC50s are compared to IC50s from the Los Alamos HIV Database (CATNAP, Compile, Analyze and Tally NAb Panels; http://hiv.lanl.gov/catnap).

## Conclusions

We have developed an efficient and easy-to-follow method to produce high quantities of sCD4 from human cells. 293 T cells can be transduced with lentiviral vectors at high MOIs. The transduced 293 T cells can be grown into a confluent monolayer and then be cultured in media with reduced serum concentration, which is a cost-effective solution to minimize serum protein contaminations in the purified protein preparations. Depending on the stability of the secreted protein, the cells can be incubated for several days before culture supernatants are harvested. Using cost-effective metal ion affinity chromatography, we have demonstrated that pure preparations of His-tagged protein can be obtained in a one-step process. We have further shown that using an alternative signal peptide could increase the protein yield. Screening potential signal peptides with the freely available software SignalP may be helpful to identify promising test candidates, but the best signal peptides would have to be experimentally determined for different proteins. The procedure should easily be adaptable for purifying other secreted proteins. For example, we have used this method to successfully produce the single chain antibody 17b in similar quantities as well as sCD4 fusion proteins, albeit at 3-fold lower concentrations. This strategy can be adapted to a large-scale format without significantly increasing the cost to further improve the yield.

## Abbreviations

AAT: Alpha-1 antitrypsin
BSA: Bovine serum albumin
CATNAP: Compile, Analyze and Tally NAb Panels
CHO: Chinese hamster ovary
CMV: Cytomegalovirus
ELISA: Enzyme-linked immunosorbent assay
Env gp120: Envelop glycoprotein 120
FBS: Fetal bovine serum
Fc: Crystalizing fragment
HEK: Human embryonic kidney
IC50s: Half maximal inhibitory concentrations
IgG: Immunoglobulin G kappa chain
MOI: Multiplicity of infection
PEI: Polyethylenimine
sCD4: Soluble CD4
TCID50: 50 % tissue culture infectious dose
TPA: Tissue plasminogen activator
WPRE: Woodchuck hepatitis post-transcriptional regulatory element
α-Gal: Galactose-α-1,3-galactose
